# Beam quality assessment: Evaluating aluminum purity's effects

**DOI:** 10.1002/acm2.70450

**Published:** 2026-01-02

**Authors:** Sara Mohammadi, Joshua Deslongchamps, Jiali Wang, Mary Ellen Jafari

**Affiliations:** ^1^ Department of Medical Imaging Technology and Informatics Southern California Permanente Medical Group Los Angeles California USA

**Keywords:** beam quality, diagnostic radiography, half‐value layer (HVL), quality assurance (QA), regulatory compliance, solid‐state detectors

## Abstract

**Purpose:**

This study evaluates the effect of aluminum (Al) purity on Half Value Layer (HVL) measurements and beam quality across mobile and fixed radiographic units. Beam quality assessment is a fundamental aspect in ensuring the safe and effective delivery of ionizing radiation to patients. Standards for this assessment vary nationally and internationally, leading to ambiguity within measurements, which may cause regulatory compliance issues.

**Methods:**

Beam filtration was measured using Al filters of 99.0% and 99.5% purity, for mobile and fixed radiographic units utilizing radiation detectors from three manufacturers. Methods and geometry followed IEC standards for HVL measurements with techniques representative of those used for standard patients. Measurements were made across a range of thicknesses of aluminum incrementally centered around the projected HVL. HVL was determined through exponential fitting of exposure versus Al thickness. Additionally, for each solid‐state detector, a single‐shot HVL measurement was performed without aluminum in the x‐ray beam, as this method is commonly used for HVL determination in clinical settings.

**Results:**

When Al filtration purity was increased from 99.0% to 99.5%, HVL measurements rose by 1.35% to 4.82%, with a median increase of 3.26% (95% confidence interval 2.68%–3.85%). The deviation between single‐shot HVL measurements and interpolated values ranged from 0.16% to 8.99% (median: 4.90%; 95% CI: 3.32%–6.49%) for 99.0% purity Al, and from 0.43% to 4.79% (median: 2.62%; 95% CI: 1.60%–3.64%) for 99.5% purity Al.

**Conclusion:**

There is a discrepancy in the purity of reference aluminum used for determining HVL specified in the United States Food and Drug Administration (FDA) and International Electrotechnical Commission (IEC) standards. FDA requires Type‐1100 aluminum with a minimum of 99.0% purity, while IEC requires a minimum 99.9% purity. Our results indicate that measured HVL values increase with aluminum purity in a magnitude sufficient to result in units falsely failing regulatory requirements for minimum HVL if different types of aluminum are used by manufacturers and physicists. Medical physicists should be aware of this issue when testing HVL for compliance with regulatory standards. This study did not provide a direct comparison of FDA and IEC standards; instead, it demonstrates the direction and magnitude of HVL sensitivity to aluminum purity within clinically realistic materials, highlighting how seemingly small variations in aluminum purity can materially influence regulatory interpretation of beam quality.

## 1 INTRODUCTION

The Half‐Value Layer (HVL) is an important parameter in the assessment of radiographic x‐ray tube performance, reflecting both the beam quality and its potential impact on patient dose. The U.S. Food and Drug Administration (FDA) mandates a minimum HVL of 2.9 mm of aluminum (Al) at 80 kV to ensure adequate filtration and safety in radiographic imaging systems.^1^ Acceptance and routine annual testing of radiographic units have revealed inconsistencies in HVL measurements, with some units failing to meet this standard when tested using the conventional narrow beam geometry method. These discrepancies have led to a comprehensive review of the HVL measurement procedures to identify potential causes.

One factor contributing to this inconsistency is the variation in aluminum purity standards between regulatory bodies. The FDA specifies Type‐1100 aluminum with a minimum purity of 99.0%, while the International Electrotechnical Commission (IEC) requires a minimum purity of 99.9% for reference aluminum used in HVL testing.^2^ This difference in material standards introduces a potential source of variability in HVL measurements, as the purity of aluminum affects x‐ray attenuation properties.

This study aims to investigate the impact of aluminum purity standards on HVL measurements and to determine if discrepancies between FDA and IEC standards contribute to observed measurement variabilities. This study does not constitute a direct FDA–IEC compliance comparison but rather demonstrates that modest increases in aluminum purity produce HVL shifts of sufficient magnitude to impact regulatory interpretation. By exploring these factors, we seek to enhance the accuracy of HVL testing methodologies and ensure compliance with regulatory requirements across radiographic units.

## 2 METHODS

A comparative study was performed using Al filters of 99.0% and 99.5% purity. Measurement using Al with 99.9% purity (as referenced by the IEC) was not possible because, although commercially available, it was not accessible for this study. Measurements were taken on both mobile and fixed radiographic systems, employing radiation measurement systems from three different manufacturers, RadCal Accu‐Gold AGDM+ (RadCal, Monrovia, California), RaySafe X2 (Fluke Corporation, Glenwood, IL), and RTI Piranha (RTI Group North America, Towaco, NJ) with Solid State Detectors (SSD). The experimental setup and methodologies adhered to IEC standards for HVL measurement, using imaging techniques representative of standard patient conditions.^2^

**FIGURE 1:**
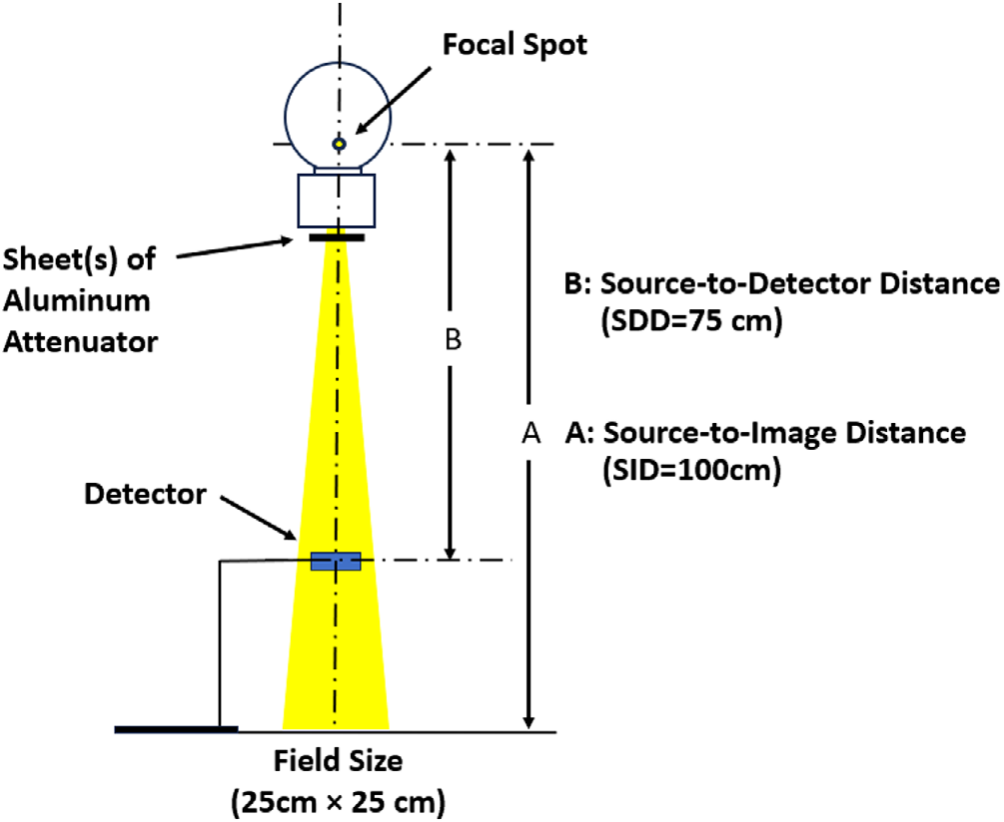
Experimental setup scheme utilizing IEC standards.

Measurements were performed on two mobile radiographic systems (Carestream DRX Revolution and Samsung GM85) and two fixed radiographic systems (Siemens Ysio Max and Philips Optimus 65). Figure 1 shows the measurement geometry. The x‐ray detector was placed in the air at 75 cm from the x‐ray source and 25 cm above the floor to reduce scatter effects. The detector was aligned at the center of the x‐ray beam, with the collimator set to a 25 × 25 cm field at a source‐to‐detector distance of 100 cm. A technique of 80 kV and 40 mAs was applied consistently across all exposures.^2^, ^3^ Because FDA HVL compliance testing is explicitly defined at 80 kV, this study focused on this regulatory reference condition rather than broader energy‐dependent beam characterization.

For each radiographic system, exposures were taken across a range of aluminum thicknesses, with incremental increases centered around the projected HVL value. Exposures were repeated using both 99.0% and 99.5% purity Al filters to assess the effect of material purity on HVL measurements. Three sequential exposures were performed for each measurement, with the average exposure value used for analysis. Using the relationship between exposure and aluminum thickness, an exponential fit was applied to the data, allowing the HVL to be determined by identifying the aluminum thickness needed to reduce the initial exposure by 50%.

A single‐shot HVL measurement was also conducted for each unit and aluminum purity level by performing exposures without any aluminum in the x‐ray beam. This single‐shot value provided a baseline for comparison against the interpolated HVL values obtained through incremental thickness measurements.

The percentage difference in HVL value between aluminum sheets of 99.0% and 99.5% purity was calculated for each radiographic unit. Additionally, deviations between the single‐shot HVL measurement and the interpolated HVL values for each aluminum purity were computed. These deviations were analyzed to assess the impact of aluminum purity on HVL measurement consistency across different radiographic models and vendors.

Sources of measurement uncertainty include detector repeatability, aluminum thickness tolerances, beam output stability, and positioning geometry. While individual uncertainty components were not independently quantified, repeated measurements and averaging were used to mitigate random variability.

## 3 RESULTS

The HVL measurement results are presented in Tables 1 and 2, revealing a notable impact of aluminum purity on HVL values in beam quality assessments. The results show HVL measurements increased by 1.35% to 4.82%, with a median increase of 3.26% (95% CI: 2.68%–3.85%) when going from 99.0% to 99.5% purity aluminum. Comparing the single‐shot value to the interpolated values with 99.0% and 99.5% aluminum, the deviation for the 99.0% aluminum ranged between 0.16%–8.99% with a median of 4.90%. The deviation for 99.5% aluminum ranged between 0.43%–4.79% with a median of 2.62%. A 4.9% deviation on a true HVL of 2.90 mm aluminum can read as low as 2.76 mm, and an 8.99% deviation could read as low as 2.64 mm. These deviations demonstrate that using higher purity aluminum results in more consistent and reliable HVL measurements, reducing the potential for apparent regulatory noncompliance.

**TABLE 1:** HVL measurements in millimeters (mm) using aluminum with 99.0% purity.

Manufacturer	Samsung GM85	Carestream DRX‐Revolution	Siemens Ysio Max	Philips Optimus 65
RadCal Ion Chamber	**2.86**	3.00	2.99	3.28
RadCal SSD	**2.87**	2.97	3.00	3.16
RaySafe SSD	2.92	3.00	3.05	3.23
Piranha SSD	**2.78**	**2.89**	2.91	3.22

Observed median percentage differences are reported in the Results with 95% confidence intervals.

**TABLE 2:** HVL measurements in millimeters (mm) using aluminum with 99.5% purity.

Manufacturer	Samsung GM85	Carestream DRX‐Revolution	Siemens Ysio Max	Philips Optimus 65
RadCal Ion Chamber	2.96	3.12	3.12	3.36
RadCal SSD	2.95	3.02	3.15	3.30
RaySafe SSD	3.00	3.04	3.16	3.38
Piranha SSD	**2.87**	2.98	3.02	3.26

Observed median percentage differences are reported in the Results with 95% confidence intervals.

**FIGURE 2:**
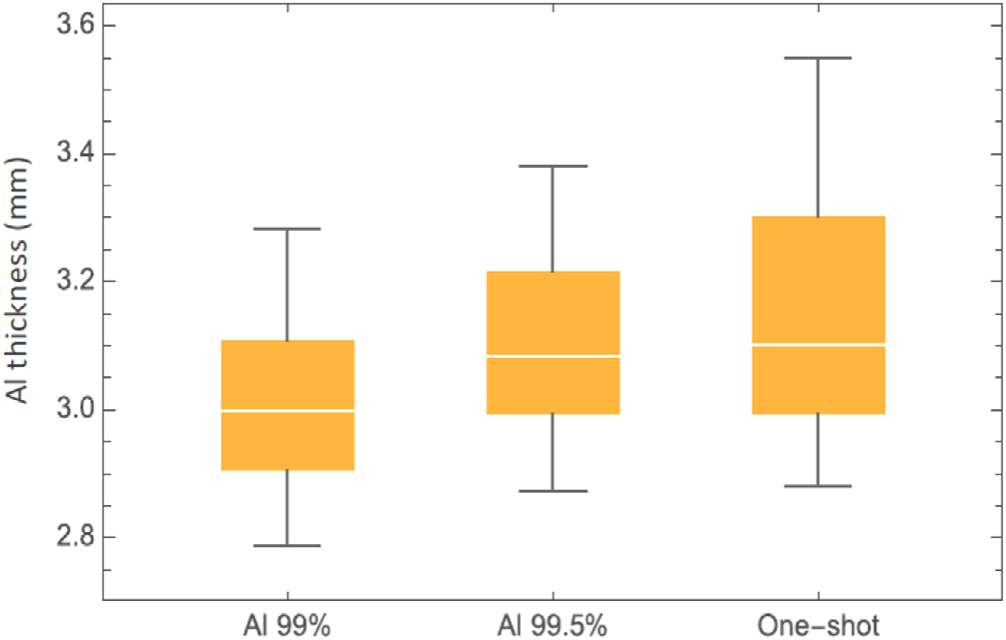
HVL measurements using aluminum with 99% and 99.5% purity compared with single‐shot values.

Figure 2 shows the HVL measurements using aluminum with 99.0% and 99.5% purity compared with single‐shot measurement values. There is a 3% difference between the single‐shot values and the 99.0% purity values, whereas the difference decreases to 1% when comparing the single‐shot values with the 99.5% purity values. These deviations would be even more substantial using 99.9% aluminum.

## 4 DISCUSSION

The findings of this study underscore the importance of standardized guidelines for HVL measurement and the need for clear specifications regarding the purity of aluminum used in beam quality assessments. The observed differences in HVL measurements between 99.0% and 99.5% purity aluminum highlight the potential for substantial variability in beam quality evaluations based on the purity of the reference material. This variability directly affects compliance and reliability in radiographic quality assurance.

The observed increase in HVL with higher‐purity aluminum filters likely arises from differences in material composition that influence x‐ray attenuation. Lower‐purity aluminum alloys contain trace elements such as silicon, iron, and copper, which have higher atomic numbers and increase photoelectric absorption of x‐ray energies. These impurities cause greater attenuation, resulting in lower measured HVL values. In contrast, high‐purity aluminum filters exhibit a more uniform electron density and lower effective atomic number, allowing x‐rays to penetrate more efficiently and yielding higher HVL readings. Moreover, higher‐purity aluminum filters typically have greater homogeneity and thickness consistency, minimizing beam hardening and improving the reproducibility of attenuation measurements.

Our results align with previous studies, which also identified the impact of aluminum purity on HVL values. Nouchi et al.^4^ demonstrated a strong linear relationship between HVL and aluminum thickness, reinforcing the finding that improved purity leads to higher HVL measurements. Similarly, Lin and Goode^5^ reported that solid‐state detectors accurately measured HVL at low tube potentials (60–80 kV) but showed inconsistencies at higher beam qualities when spectral shaping filters were used. This variability is consistent with our observation that single‐shot HVL measurements can differ appreciably from interpolated methods, emphasizing the need to evaluate the accuracy of single‐shot methodologies in clinical settings.

The discrepancies in HVL measurements due to differences in aluminum purity may have important regulatory implications, particularly in regions that follow divergent standards. For example, radiographic units manufactured to meet IEC testing standards may fail to meet FDA standards if the effect of aluminum filtration purity is not taken into account in clinical HVL measurements. Such differences could lead to confusion in equipment certification, inconsistencies across regulatory compliance, and potential safety concerns if beam quality is inaccurately assessed. For example, a radiographic unit that passes IEC‐based compliance testing using 99.9% purity aluminum could appear to fail FDA‐based testing if evaluated using 99.0% aluminum, as the measured HVL may decrease. This discrepancy could result in unnecessary corrective actions or equipment rejection during quality assurance audits. Similarly, physicists performing acceptance testing with non‐standard aluminum filters may inadvertently document HVL values below the minimum regulatory threshold (2.9 mm Al at 80 kV), prompting false reports of non‐compliance despite the equipment operating within acceptable limits. These findings highlight the need for consistent specification of aluminum purity in both manufacturing and regulatory contexts. This emphasizes the need for harmonized international standards or, at a minimum, explicit recognition of aluminum purity specifications within regional guidelines.

The variance observed in single‐shot HVL values also raises questions about the assumptions regarding aluminum purity in their calculation. As highlighted by Lin and Goode,^5^ solid‐state detectors can produce varying results depending on calibration and setup, suggesting that single‐shot methods may introduce measurement errors if aluminum purity is not adequately controlled. This issue warrants further investigation to better understand the impact of single‐shot methodology on HVL accuracy. This is a topic for future research that could inform refinements in HVL testing practices and regulatory standards.

One of the primary limitations of this study is the lack of aluminum (Al) with 99.9% purity, as recommended by the IEC. Although this level of purity is commercially available, it was not accessible for this study. Consequently, the study was conducted using aluminum filters of 99.0% and 99.5% purity. This limitation restricts the ability to directly compare the results with the IEC‐recommended standard, potentially affecting the accuracy and generalizability of the findings. Accordingly, any conclusions regarding IEC‐based compliance are inferential rather than experimental, and the present findings should be interpreted as indicating the potential impact of aluminum purity on regulatory assessment, rather than as a direct FDA–IEC comparison. Future studies should consider including 99.9% purity aluminum to enable a more precise evaluation of beam quality and facilitate direct comparisons with established standards. Results may differ at other tube potentials, particularly above 100 kV.

## 5 CONCLUSION

This study demonstrates that aluminum purity substantially impacts HVL measurements, influencing the assessment of beam quality in radiographic units. The differences in HVL measurements observed between 99.0% and 99.5% purity aluminum underscore the potential for variability in beam quality evaluations based on the reference material's purity. Such variability can directly affect compliance and reliability in radiographic quality assurance, potentially leading to units erroneously failing HVL assessments due to the purity of aluminum used by vendors, physicists, or engineers during testing.

We recommend the development of more stringent and harmonized guidelines for HVL measurement to address these discrepancies. Specifically, regulatory organizations should provide clear specifications regarding the required purity of aluminum for beam quality assessments, ensuring that radiographic units are evaluated consistently across different regions and standards. This consistency would reduce ambiguity in compliance and improve the reliability of HVL measurements, ultimately enhancing patient safety and the quality of radiographic imaging practices.

## AUTHOR CONTRIBUTIONS

All authors contributed substantially to and participated in this study.

## CONFLICT OF INTEREST STATEMENT

The authors declare no conflicts of interest.
